# A randomised controlled trial of a psychoeducational intervention for women at increased risk of breast cancer

**DOI:** 10.1038/sj.bjc.6601519

**Published:** 2004-01-06

**Authors:** S Appleton, M Watson, R Rush, S Garcia-Minaur, M Porteous, J Campbell, E Anderson, A Cull

**Affiliations:** 1 Cancer Research UK, Edinburgh Oncology Unit, Western General Hospital, Crewe Road South, Edinburgh EH4 2XR, UK; 2 Department of Psychological Medicine, The Royal Marsden NHS Trust and Institute of Cancer Research, Down's Road, Sutton, Surrey, SM2 5PT, UK; 3 Department of Clinical Genetics, Molecular Medicine Centre, Western General Hospital, Crewe Road South, Edinburgh EH4 2XU, UK; 4 Edinburgh Breast Unit, Western General Hospital, Crewe Road South, Edinburgh EH4 2XU, UK

**Keywords:** psychoeducational intervention, written information, increased risk, familial breast cancer

## Abstract

This study aimed to compare the impact of two versions of a psychoeducational written intervention on cancer worry and objective knowledge of breast cancer risk-related topics in women who had been living with an increased risk of familial breast cancer for several years. Participants were randomised to three conditions: scientific and psychosocial information pack (Group 1), scientific information pack only (Group 2) or standard care control (Group 3). They completed postal questionnaires at baseline (*n*=163) and 4 weeks (*n*=151). As predicted, there was a significant decrease in cancer worry for Group 1, but not Group 2. Objective knowledge significantly improved for both Group 1 and Group 2 as expected, but not Group 3. However, there was an unpredicted decline in cancer worry for Group 3. This study supports the value of a scientific and psychosocial information pack in providing up-to-date information related to familial risk of breast cancer for long-term attendees of a familial breast cancer clinic. Further research is warranted to determine how the information pack could be incorporated into the existing clinical service, thus providing these women with the type of ongoing psychosocial support that many familial breast cancer clinics are currently lacking.

In recent years, growing numbers of women with a family history of breast cancer have sought genetic risk counselling to gain information about their risk of developing the disease. This increase in public awareness of family history as a risk factor for breast cancer results from scientific advances in understanding cancer genetics and growing media attention to breast cancer. Genetic testing for breast cancer susceptibility was initially expected to be widely available and informative for all women identified as being at increased risk due to family history. However, it is apparent that at present genetic testing is not an option for the majority of these women for whom a mutation in a known breast cancer susceptibility gene has not been identified in an affected relative. In the absence of proven methods to prevent or reduce the risk of breast cancer, the clinical management of these women is focused primarily on breast cancer screening.

Large numbers of women under the age of 50 years are currently attending familial breast cancer clinics on a regular basis for clinical surveillance. As these women are faced with multiple chronic uncertainties regarding their personal risk of developing breast cancer and the effectiveness of risk management, the potential for adverse psychosocial effects is clear. However, psychosocial support or educational information out with regular breast cancer screening appointments (unless specifically requested) are not routinely provided for long-term attendees of many familial breast cancer clinics, including the South East Scotland familial breast cancer clinic in Edinburgh.

There is limited psychosocial research in women who have been living with an increased risk of familial breast cancer for at least 2 years (Appleton, 2003; Appleton *et al*, 2000). Although the prevalence of ‘case-level’ general psychological distress is similar to that found in the general population, levels of breast-cancer-specific distress are higher and there is a high prevalence of worries about breast cancer risk-related issues (Appleton, 2003). This study highlighted a widespread need among these women for up-to-date, reliable scientific and psychosocial information related to familial risk of breast cancer with an overall preference for the information to be presented in a written format.

A number of different psychological interventions have been evaluated in women with a family history of breast cancer (e.g. Gagnon *et al*, 1996; Lerman *et al*, 1996; Cull *et al*, 1998; Schwartz *et al*, 1998; Watson *et al*, 1998; Audrain *et al*, 1999; Kash *et al*, 1999; Wellisch *et al*, 1999; Esplen *et al*, 2000). The studies of particular interest are those that have investigated the impact of psychoeducational group interventions in American women at high risk of breast cancer (i.e. Kash *et al*, 1999; Wellisch *et al*, 1999). Wellisch *et al* (1999) carried out a pilot study of a 6-week group intervention to treat psychological distress in 33 women enrolled in a high-risk surveillance programme. The weekly group meetings consisted of educational (e.g. genetics) and psychological components (e.g. share experiences). Although statistically significant reductions in depression and state anxiety were observed, there are a number of methodological limitations to the study that the authors acknowledge (e.g. small sample size, short-term follow-up only and lack of control group). Kash *et al* (1999) reported preliminary results of a randomised controlled trial of a 1-year psychoeducational group intervention in 192 women at high risk of breast cancer. The intervention consisted of education, social support enhancement, problem-solving and cognitive restructuring. Women in the intervention group experienced a statistically significant decrease in breast-cancer-specific anxiety and perceived risk and improvement in knowledge between baseline and 1-year follow-up.

Given the need for information and the prevalence of worry in 249 British women living with an increased risk of familial breast cancer (Appleton, 2003), a psychoeducational written intervention was developed, described in detail elsewhere (Appleton *et al*, 2003).

The present study aimed to determine the impact of two versions of this intervention on cancer worry (primary outcome) and objective knowledge of breast cancer risk-related topics (secondary outcome). The subsidiary aims were: to explore the impact of the interventions on breast-cancer-specific distress, general psychological distress and appraisal (i.e. perceived risk and perceived control over developing breast cancer); to evaluate the acceptability of the interventions.

Three hypotheses were tested:

- *Scientific and psychosocial* written information will reduce cancer worry to a greater extent than *scientific* written information alone.
- *Scientific* written information will reduce cancer worry to a greater extent than *standard care* alone (i.e. the regular clinical surveillance provided by the familial breast cancer clinic in accordance with the Scottish Cancer Group & Cancer Genetics Sub-Group (2001) clinical guidelines based on age and family history).
- *All* written information will improve objective knowledge of breast cancer risk-related topics to a greater extent than *standard care* alone.

## MATERIALS AND METHODS

### Design

A randomised controlled trial comparing three groups:

Group 1: Scientific and psychosocial information pack.

Group 2: Scientific information pack.

Group 3: Standard care only (control group).

All three groups were assessed at baseline (prior to receiving the information pack) and postintervention (approximately 4 weeks later) by postal questionnaire.

### Participants

Ethical approval for the study was obtained from the local ethics committee. Women who had received breast cancer genetic risk counselling were recruited using a database for South East Scotland held in the Department of Clinical Genetics, Western General Hospital, Edinburgh. Women were included if they were currently enrolled at the Ardmillan Familial Breast Cancer Clinic, had attended the clinic for at least 2 years and had indicated in an earlier questionnaire study (Appleton, 2003) that they were interested in at least one of the intervention options listed. Women were excluded if they had a previous diagnosis of cancer, prophylactic surgery, genetic testing or were currently participating in the International Breast Cancer Intervention Study (IBIS), Magnetic Resonance Imaging (MRI) Trial or the Cancer Genetics in the Community Trial. At the point of recruitment to the previous study (Appleton, 2003), GPs had excluded women who were suffering from serious physical illness, alcoholism, schizophrenia or organic brain damage. All women meeting the entry criteria were invited to participate.

### Sample size calculation

There is a lack of data to suggest what constitutes a clinically significant change on the Cancer Worry Scale (CWS). Therefore, the sample size calculations were based on an effect size of 0.5 which is generally regarded as moderately large (Cohen, 1988, pp 291) and a s.d. of scores on the CWS of 2.58 from a sample of 116 women at increased risk of breast cancer (G Rees, 2000, personal communication). The mean change in scores (0.5 × 2.58=1.3) was rounded up to 1.5 to allow for greater variability in CWS scores in this sample. To detect a difference of 1.5 on the CWS with an 80% power at a significance level of 5%, a minimum sample size of 138 (i.e. 46 women in each of the three groups) was required.

### Psychoeducational intervention

The intervention was a psychoeducational written information pack consisting of 10 scientific and psychosocial topics of information related to familial risk of breast cancer (i.e. 1. Introduction to breast cancer, 2. Breast cancer genetics, 3. Genetic testing, 4. Options for women with a family history of breast cancer, 5. Hormone replacement therapy (HRT), 6. Diagnosis and treatment of breast cancer, 7. Research at the Ardmillan familial breast cancer clinic, 8. Healthy lifestyle, 9. Worry about breast cancer, 10. Sources of information) and three published leaflets to accompany topics 3, 4 and 9 (i.e. ‘Cancer genetics’, South East of Scotland Clinical Genetics Service, 2001; ‘Breast awareness’, Breast Cancer Care, 2000; ‘How to… stop worrying’, MIND, 1998).

Two versions of the information pack were evaluated: the scientific and psychosocial information pack (which contained all topics and leaflets) and the scientific information pack (which contained all topics and leaflets except the two psychosocial topics, ‘Healthy lifestyle’ and ‘Worry about breast cancer’ and accompanying leaflet ‘How to…stop worrying’). The development and content of the intervention has been described in more detail elsewhere (Appleton *et al*, 2003).

### Sociodemographic and objective breast cancer risk characteristics

Several sociodemographic characteristics of the women were assessed including: age, education, marital status, occupation, number of children, number of years attendance at the familial breast cancer clinic and current objective breast cancer risk (as assessed by a specialist registrar, S G-M and a consultant in clinical genetics, MP). Objective breast cancer risk was classified as: low (<17% lifetime risk), medium low (17–19%), medium (20–22%), medium high (23–25%) and high (>25%).

### Psychological measures

#### Cancer Worry Scale (Watson *et al*, 1998)

This six-item scale (adapted from four single items, Lerman *et al*, 1991a, 1991b, 1993, 1994) assesses concerns about developing cancer and the impact of cancer worry on daily functioning in terms of its frequency and severity. Responses are on a four-point Likert scale and are summed to produce a total score of 6–24, where a higher score indicates higher levels of worry.

#### Objective knowledge of breast cancer risk-related topics

A study-specific measure of 36 items assessing objective knowledge of the key points of information covered in the scientific topics of the information pack. This consisted of 17 items on breast cancer genetics and genetic testing (e.g. ‘Most women diagnosed with breast cancer: carry an inherited genetic mistake?’), 14 items on breast cancer screening (e.g. ‘Mammography: can prevent breast cancer?’) and five items on HRT (e.g. ‘For women with a family history of breast cancer: the effect of using HRT on the risk of breast cancer is not clear?’). Participants were asked to respond ‘true’, ‘false’ or ‘don't know’. The number of correct, incorrect and ‘don't know’ responses were summed separately to produce three total scores all ranging from 0 to 36.

#### Impact of event scale (Horowitz *et al*, 1979)

This 15-item scale determines levels of intrusive and avoidant thoughts about breast cancer risk in the past week (Kash *et al*, 1992). Responses are on a four-point Likert scale that are assigned weighted scores (0,1,3,5). Scores are summed to produce two subscale scores and a total score: intrusion (0–35); avoidance (0–40); total (0–75), where higher scores represent greater breast-cancer-specific distress. An opt-out box used in previous research (i.e. Lloyd *et al*, 1996) was included for women who had not thought about their risk of breast cancer in the past week.

#### General health questionnaire 12-item version (GHQ-12) (Goldberg and Williams, 1988)

This well-validated first-stage screening test was scored using the GHQ method (0, 0, 1, 1) using a threshold of *⩾*3 to screen for ‘case-level’ general psychological distress.

#### Perceived risk

A single item was used to measure perceived likelihood of developing breast cancer: ‘How likely do you feel it is that you will ever develop breast cancer?’. Responses were on a five-point Likert scale (very unlikely/unlikely/likely/very likely/inevitable).

#### Perceived control

A single item was used which has been developed to assess perceived control over developing breast cancer in women at increased risk (‘How much control do you feel you have over whether you develop breast cancer?’) (Audrain *et al*, 1997). Responses were on a four-point Likert scale (none at all/a bit/moderate/a lot).

#### Evaluation of the intervention

Several items were used to obtain feedback from participants in Groups 1 and 2 postintervention on the information pack in terms of:

- the number of times they read the topics/leaflets,
- when they last read any of the information pack,
- to what extent the information included in each topic was new to them,
- if they discussed or gave the information pack to anyone else,
- if they found any topics/leaflets difficult to understand, upsetting or helpful,
- if they had changed or intend to change any of their health behaviours as a result of reading the information pack,
- if they intended to obtain any of the further reading listed,
- if they thought any topics were missing from the pack,
- if the information pack covered their need for information and support.

#### Additional information

Participants in Group 3 were asked postintervention if they had read any information related to familial risk of breast cancer in the past month.

### Procedure

Potential participants were sent an information sheet and consent form which they were asked to return to indicate whether they were willing to participate in the trial. Women who consented to participate were sent a baseline postal questionnaire and letter notifying them to which of the three groups they had been randomised. Restricted randomisation using the random permuted blocks method (Pocock, 1983, pp 76–79) was undertaken to ensure that there were equal numbers of participants in each of the three groups. On return of the completed baseline questionnaire, participants in Groups 1 (scientific and psychosocial information) and 2 (scientific information) were sent the appropriate information pack. Postintervention questionnaires were sent to participants 4 weeks after sending them an information pack (Groups 1 and 2) or 4 weeks after they returned a completed baseline questionnaire (Group 3). At the end of the postintervention questionnaire, Groups 2 and 3 were offered the full written information pack (i.e. Group 2: psychosocial topics, Group 3: scientific and psychosocial topics) and were sent it immediately if requested. The GP was promptly notified by letter if their patient scored above the clinical case threshold (i.e. *⩾*3) on the GHQ-12 at either baseline or postintervention (participants having given their consent on recruitment to the study).

### Statistical methods

Descriptive statistics were used to describe participants and to summarise feedback on the information pack. Differences between two groups (e.g. participants and nonparticipants) were analysed with independent samples *t*-tests (two-tailed), Mann–Whitney tests or χ^2^ tests (two-tailed). Comparisons of the three groups at baseline and postintervention were made using the Kruskal–Wallis test, the χ^2^ test (two-tailed) or one-way ANOVA. Changes between baseline and postintervention were assessed for each of the three groups (only for women with data at both assessments) by the Wilcoxon matched-pairs signed-rank test or the McNemar test. A number of categorical variables were recoded for the purposes of between- and within-group analysis: breast cancer risk (low or medium low/medium/medium high or high), perceived likelihood of developing breast cancer (unlikely/likely), perceived control over developing breast cancer (no control/some control), marital status (married or living with a partner/not married or living with a partner) and occupation (employed/not employed). The absence of one or more scores on a scale resulted in that total score being classified as missing. Data were analysed on an intention-to-treat basis. A significance level of 0.05 was used throughout. The data were analysed using SPSS for Windows version 10.00 (1999).

## RESULTS

### Participants

Figure 1

**Figure 1:**
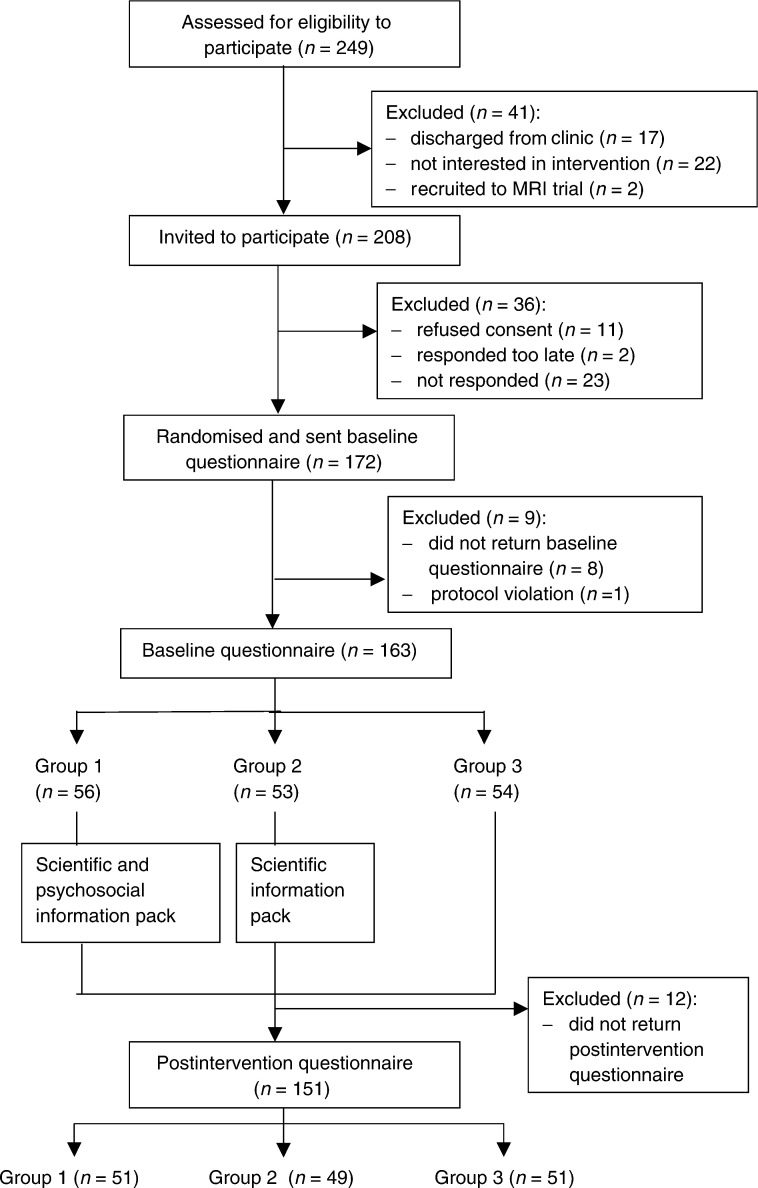
Progress of participants through the trial.

shows the progress of participants through the trial.

#### Baseline

Of the 208 women eligible to participate in the trial, 11 refused, two responded too late to be randomised (3 months after the initial invitation) and 23 did not reply. Of the 172 women who consented to participate, were subsequently randomised and sent the baseline questionnaire, eight (from Group 1=one; Group 2=four; Group 3=three) did not return the questionnaire and one woman (randomised to Group 3) was excluded from the analysis because of a protocol violation (she had been discharged from the clinic prior to randomisation and therefore should not have been invited to participate). Therefore, 163 baseline questionnaires were included in the analysis. There were no significant differences between the participants at baseline (*n*=163) and nonparticipants (*n*=45) on any of the sociodemographic or objective breast cancer risk characteristics.

#### Postintervention

In total, 12 women (from Group 1=five; Group 2=four; Group 3=three) dropped out of the study between baseline and postintervention (i.e. they did not return the postintervention questionnaire). A total of 151 women (73% of the 208 eligible women) completed both questionnaires. The number of weeks between completing the baseline and postintervention questionnaires ranged from 4.71 to 17.57 (mean=7.51, s.d.=2.5). Differences between participants who only completed the baseline questionnaire (*n*=12) and those who completed both questionnaires (*n*=151) could not be tested statistically due to the small sample size. The majority of participants in Groups 2 and 3 postintervention requested to be sent the full information pack (Group 2: *n*=35, 71%, Group 3: *n*=48, 94%).

### Sociodemographic and objective breast cancer risk characteristics

Participants ranged in age from 28–62 years (mean=43.9 years, s.d.=6.57). They had been attending the Ardmillan Familial Breast Cancer Clinic for 2.75–8.51 years (mean=5.26, s.d.=1.72). The majority of participants were married or living with a partner (*n*=137, 86%), had at least one child (*n*=131, 81%) and were employed (*n*=135, 83%). A total of 53 women (33%) had received schooling until age 16 years only whereas 27 (17%) had attended school/further education/training until age 18 years , 38 (24%) had further education or training after age 18 years and 44 (27%) were university graduates. The majority were estimated to be at medium risk of breast cancer (*n*=80, 53%) with 41 (27%) assigned a medium high risk, 15 (10%) medium low, nine (6%) low (these women were likely to soon be discharged from the clinic) and five (3%) high. Current objective breast cancer risk was not assessed for 13 participants, as their clinical case notes could not be located. There were no significant differences between the three groups at baseline on any of the sociodemographic or objective breast cancer risk variables.

### Psychological characteristics: comparison of the three groups and changes over time within groups

Table 1

**Table 1:** Psychological characteristics of the three trial groups at baseline and postintervention

	**Baseline (*n*=163)**			**Postintervention (*n*=151)**		
	**Group 1**	**Group 2**	**Group 3**	**Group 1**	**Group 2**	**Group 3**
**Variable**	**(*n*=56)^b^**	**(*n*=53)^c^**	**(*n*=54)^d^**	**(*n*=51)**	**(*n*=49)**	**(*n*=51)**
Cancer Worry Scale: median (range)	9 (6–20)	9 (6–17)	10 (6–21)	9 (6–16)	9 (6–24)	9 (6–16)
Objective knowledge: median (range)^e^						
Total correct	17.5 (6–28)	17 (8–35)	15(7–31)	24 (3–33)	27 (11–36)	15 (5–33)
Total incorrect	9 (3–18)	8(1–19)	8 (1–15)	8 (2–18)	7 (0–13)	9 (2–17)
Total ‘don't know’	9 (0–23)	10.5 (0–21)	12 (0–22)	2 (0–26)	1 (0–16)	12.5 (0–25)
Impact of Event Scale: median (range)^f^						
Intrusion	8 (0–33)	6 (0–25)	5 (0–27)	9 (0–13)	11(0–35)	3.5 (0–25)
Avoidance	9 (0–26)	8 (0–23)	9 (0–30)	10.5 (0–29)	16 (0–40)	5.5 (0–30)
Total score	18 (0–59)	12.5 (0–47)	13 (0–51)	18 (0–42)	30 (0–75)	8.5 (0–51)
GHQ-12:						
Total score: median (range)	1 (0–11)	0 (0–12)	0 (0–12)	0 (0–11)	0 (0–12)	0 (0–10)
‘Case-level’ distress: *n* (%)^g^	17 (30%)	10 (20%)	20 (37%)	7 (14%)	7 (14%)	12 (25%)
Perceived likelihood of developing						
breast cancer: *n* (%)						
Unlikely	15 (27%)	13 (26%)	17 (33%)	16 (34%)	19 (41%)	14 (28%)
Likely	41 (73%)	38 (75%)	35 (67%)	31 (66%)	27 (59%)	36 (72%)
Perceived control over developing						
breast cancer: *n* (%)						
None at all	18 (32%)	21 (40%)	16 (30%)	6 (12%)	14 (29%)	18 (35%)
Some	38 (68%)	32 (60%)	38 (70%)	44 (88%)	34 (71%)	33 (65%)

a Sample size varies due to missing data.

b Group 1 received the scientific and psychosocial information pack.

c Group 2 received the scientific information pack.

d Group 3 received standard care only (control group).

e Possible range of scores: 0–36.

f A total of 71 participants (44%) at baseline and 52 (35%) postintervention indicated that they had thought about the risk of breast cancer in the past week and therefore completed the Impact of Event Scale.

g Scores of ⩾3.

compares the psychological characteristics of the three groups at baseline (where there were no significant differences between groups) and postintervention.

#### Cancer worry

Groups 1 (*z*=−2.133, *P*=0.033) and 3 (*z*=−2.449, *P*=0.014) only showed a significant decrease in scores on the CWS from baseline to postintervention. Examination of changes in individual items of the CWS for Group 3 indicated that the only item to have improved to a level approaching significance was ‘during the past month, how often have you thought about your own chances of developing cancer?’ (*P*=0.057).

#### Objective knowledge of breast cancer risk-related topics

There were significant differences between the three groups postintervention on objective knowledge: total correct (*χ*^2^=37.387, df=2, *P*=0.000), objective knowledge: total incorrect (*χ*^2^=6.760, df=2, *P*=0.034), objective knowledge: total don't know (*χ*^2^=37.487, df=2, *P*=0.000). In Groups 1 and 2 only, there were significant changes between baseline and postintervention on the objective knowledge total scores. In both groups, there was a significant increase in the total number of correct responses (*z*=−4.605, *P*=0.000; *z*=−5.090, *P*=0.000) and a significant decrease in the number of answers participants didn't know (*z*=−4.579, *P*=0.000; *z*=−5.000, *P*=0.000). In Group 2 only, there was also a significant decrease in the total number of incorrect responses (*z*=−2.210, *P*=0.027). Examination of the individual knowledge items revealed persistent misunderstandings in Group 1 where over half of the group postintervention still gave the incorrect response (e.g. ‘The following are designed to reduce the risk of breast cancer developing: mammography?’).

#### Other psychological variables

There was a significant difference between the three groups postintervention on perceived control (*χ*^2^=7.711, df=2, *P*=0.021). For Group 1, there was a significant increase in perceived control from baseline to postintervention (*P*=0.004). Group 2 experienced a significant decrease in perceived likelihood of developing breast cancer (*P*=0.039). In Group 3, there was a significant decrease in scores on the intrusion subscale (*z*=−2.248, *P*=0.025), but no significant changes on avoidance and total Impact of Event Scale scores.

### Evaluation of the intervention

In total, 80% of the women in Group 1 and 89% of Group 2 had read all of their information pack. Only two women (both in Group 1) had not read any of their information pack. There were no significant differences between Groups 1 and 2 on when they had last read any of the information pack with most women reading it more than 2 weeks ago (*n*=47, 47%). The majority of participants in both groups (60–95%) thought that the information included in every topic was at least ‘a little’ new to them. A total of 45% of women in Group 1 (*n*=23) and 27% in Group 2 (*n*=13) had discussed the information in their pack with somebody else (e.g. husband, sister, daughter). Nine women (18%) in Group 1 and seven (14%) in Group 2 stated that someone else had read their information pack. Only 12 participants (12%) found any of the topics difficult to understand that were most frequently ‘breast cancer genetics’ and ‘genetic testing’. Similarly, only five women (5%) found any of the topics of information upsetting (e.g. ‘diagnosis and treatment of breast cancer’). Participants generally found all of the topics of information and leaflets helpful: 23–45% rated each topic as ‘very much helpful’ and 48–54% rated each leaflet as ‘quite a bit helpful’. The majority of participants reported changing or intending to change certain health behaviours since reading the information pack: greater breast awareness (*n*=45, 47% of Groups 1 and 2); healthier lifestyle (*n*=24, 50% of Group 1); use of techniques to relieve worries about breast cancer (*n*=26, 57% of Group 1). A total of 27% of participants (*n*=24) thought that they would obtain some of the further reading listed in the information pack. Only one woman in Group 1 (1%) and five women in Group 2 (10%) thought that there was any information not included in the pack that they would have liked to know (e.g. more details on genetic testing and new cancer treatments). A total of 92% of Group 1 (*n*=44) and 98% of Group 2 (*n*=48) thought that the information pack covered their need for information and support.

### Additional information

Postintervention, only two women in Group 3 (4%) had read any information related to familial risk of breast cancer in the past month (i.e. about breast awareness and genetic testing).

## DISCUSSION

This study evaluated the impact of a psychoeducational intervention on cancer worry and knowledge in women living with an increased risk of breast cancer. Compliance with the study was good as the participation rate for completing both questionnaires was 73%.

The findings of the study provide evidence to support the first hypothesis. There was a statistically significant decrease in the CWS scores of Group 1 from baseline to postintervention and no corresponding decrease in Group 2. This suggests that the psychosocial topics played a key role in the reduction of cancer worry. However, further research would be needed to identify which specific information made the greatest contribution to this reduction. The results contrast those of a previous study in American women attending breast cancer genetic risk counselling where a written newsletter in addition to counselling was not shown to reduce breast-cancer-specific distress to a greater extent than counselling alone (Gagnon *et al*, 1996).

The results of this study do not support the second hypothesis as there was a significant decrease in CWS scores for Group 3 (the control group), but not for Group 2. This finding may be due to the control group not being contacted about the study for 4 weeks after returning the baseline questionnaire, which meant they may have experienced fewer cues to remind them about breast cancer. Indeed, the only CWS item to have decreased to a level of near statistical significance was the frequency they had thought about their own chances of developing cancer in the past month. Research has described the experiences of women at increased risk of breast cancer concerning increased anxiety prompted by a variety of breast cancer cues (Appleton *et al*, 2000).

There was a considerable amount of support for the third hypothesis. Objective knowledge of breast cancer risk-related topics significantly improved in Group 1 (i.e. more correct responses, fewer ‘don't know’ responses) and Group 2 (i.e. more correct responses, fewer incorrect responses and fewer ‘don't know’ responses), but remained unchanged in Group 3. Although there was a slight decrease in the number of incorrect responses of Group 1 between baseline and postintervention, it was not found to be statistically significant. As this group was sent more material to read and absorb than Group 2, it is possible that the full information pack was too lengthy to be effectively retained. This is also reflected in the fact that Group 2's knowledge seemed to improve to a greater extent than Group 1. Although Group 1 did not provide any feedback to suggest there was too much information in the pack, a smaller proportion of the women in Group 1 reported reading the whole of the information pack than Group 2. Future research could investigate the effectiveness of written information of varying detail and length to enable the development of an intervention that is of optimal benefit to these women. The persistent misunderstandings of Group 1 where over half of the group still gave the incorrect response postintervention were concerning genetic testing and screening. These findings highlight areas where the information pack could perhaps have provided more emphasis and where future interventions may choose to focus. Persistent errors in understanding of certain issues were reported in British first-time attendees of genetic risk counselling for breast cancer, despite receiving a video about breast cancer genetics and screening (Cull *et al*, 1998).

In addition to the key outcomes, the psychoeducational written intervention was also shown to affect other psychological variables. The significant increase in perceived control over ever developing breast cancer in Group 1 highlights the potential value of providing self-help psychosocial information. These results support previous qualitative findings where women with an increased risk of breast cancer described how adopting a healthier lifestyle had enhanced their feelings of control over their risk of breast cancer (Appleton *et al*, 2000). The increase in perceived control may also help to explain the decrease in cancer worry in Group 1. It has been suggested that ‘low levels of perceived control may increase vulnerability to cancer-specific distress’ in women with a family history of breast/ovarian cancer (Audrain *et al*, 1997). Taylor *et al* (1984) has shown that in breast cancer patients perceived control over the disease (both in terms of internal and external control) was significantly associated with good adjustment to breast cancer. In Group 2, the significant decrease in the perceived likelihood of ever developing breast cancer may be due to an improvement in the accuracy of their perceived risk. Further research would be needed to investigate the role of the accuracy of risk perception both as a moderator and outcome of psychoeducational intervention. The significant decrease in intrusive thoughts about breast cancer risk in Group 3 mirrors the decrease in cancer worry in this group and again may be due to the fact that the group was not contacted about the study during the 4 weeks between questionnaire assessments.

Both versions of the information pack were generally found to be highly acceptable to participants and to meet their needs for information and support. The impact of the information packs on changes in or intention to change particular health behaviours was encouraging since behavioural change was not the main focus of this brief intervention. However, these results should be interpreted with caution as the social desirability of indicating an improvement in health behaviour was not assessed and an intention to change a particular health behaviour is not necessarily realised (Marteau and Lerman, 2001). Therefore, further research could investigate the addition of a follow-up intervention to help participants realise their good intentions.

There are several methodological limitations of the study that should be considered. As the data were analysed on an intention-to-treat basis, data from the minority of women who had not read all of their information pack and the two women who had not read any of their information pack were retained in the analysis. Therefore, the reduction in cancer worry and improvement in knowledge are likely to be slightly conservative. At baseline, the median score on the CWS for each group (Group 1=9; Group 2=9; Group 3=10) was slightly lower than for those reported in women prior to attending breast cancer genetic risk counselling (Watson *et al*, 1998: median=11; Hopwood *et al*, 2001: median=11) and 2–21 months postcounselling (Hopwood *et al*, 2001: median=11). Due to the lack of clinical thresholds on the CWS, it was difficult to determine the exact clinical significance of this baseline level of cancer worry and the postintervention reduction in cancer worry. However, the majority of participants reported that the ‘Worry about breast cancer’ topic was helpful and many reported using or intending to use the suggested techniques to relieve their worry. Further work is warranted to identify CWS clinical thresholds to aid the identification of worried individuals and to measure the clinical effectiveness of associated interventions. In addition, further development of the objective knowledge measure is required including psychometric testing and reference data to indicate the clinical significance of improvements. The number of participants in each group was relatively small, they were recruited from one familial breast cancer clinic and were self-selected as they had all expressed an interest in at least one of the intervention options listed in a previous study (Appleton, 2003). A large multicentre trial would be warranted to confirm the value of the psychoeducational intervention among a wider population of women living with an increased risk of breast cancer. In addition, it would be desirable to incorporate a long-term follow-up to discover if any of the short-term improvements in the key outcomes are sustained.

This study supports the value of a psychoeducational written intervention in providing up-to-date information related to familial risk of breast cancer for long-term attendees of a familial breast cancer clinic. The scientific and psychosocial information pack reduced cancer worry and improved knowledge while meeting the subjective needs of these women. Further investigation is warranted to: undertake an economic evaluation of the intervention to determine whether it would be a cost-effective addition to the clinical service; determine how the information pack could be incorporated into the existing clinical service, thus providing these women with the type of ongoing psychosocial support that many familial breast cancer clinics are currently lacking.

## References

[bib1] Appleton S (2003) Cross-sectional study to investigate the impact of appraisal, coping style, social support and breast cancer cues on psychological distress in women living with an increased risk of breast cancer (Chapter 4). Psychosocial effects of living with an increased risk of breast cancer, Unpublished PhD Thesis, University of Edinburgh

[bib2] Appleton S, Fry A, Rees G, Rush R, Cull A (2000) Psychosocial effects of living with an increased risk of breast cancer: an exploratory study using telephone focus groups. Psycho-Oncology 9: 511–521 11180586 10.1002/1099-1611(200011/12)9:6<511::aid-pon469>3.0.co;2-e

[bib3] Appleton S, Garcia-Minaur S, Porteous M, Campbell J, Anderson E, Watson M, Cull A (2003) The development of a psychoeducational intervention for women living with an increased risk of breast cancer. Patient Educ Couns (in press) 10.1016/j.pec.2003.08.004 15476996

[bib4] Audrain J, Rimer B, Cella D, Stefanek M, Garber J, Pennanen M, Helzlsouer K, Vogel V, Hsiang Lin T, Lerman C (1999) The impact of a brief coping skills intervention on adherence to breast self-examination among first- degree relatives of newly diagnosed breast cancer patients. Psycho-Oncology 8: 220–229 10390734 10.1002/(SICI)1099-1611(199905/06)8:3<220::AID-PON370>3.0.CO;2-C

[bib5] Audrain J, Schwartz M, Lerman C, Hughes C, Peshkin B, Biesecker B (1997) Psychological distress in women seeking genetic counseling for breast-ovarian cancer risk: the contributions of personality and appraisal. Ann Behav Med 19(4): 370–377 9706364 10.1007/BF02895156

[bib6] Cohen J (1988) Statistical Power Analysis for the Behavioral Sciences, pp 291, Erlbaum: Hove

[bib7] Cull A, Miller H, Porterfield T, Mackay J, Anderson EDC, Steel CM, Alton RA (1998) The use of videotaped information in cancer genetic counselling: a randomized evaluation study. Br J Cancer 77(5): 830–837 9514066 10.1038/bjc.1998.135 PMC2149970

[bib8] Esplen M, Toner B, Hunter J, Glendon G, Liede A, Narod S, Stuckless N, Butler K, Field B (2000) A supportive-expressive group intervention for women with a family history of breast cancer: results of a phase II study. Psycho-Oncology 9: 243–252 10871720 10.1002/1099-1611(200005/06)9:3<243::aid-pon457>3.0.co;2-i

[bib9] Gagnon P, Massie MJ, Kash KM, Gronert M, Heerdt AS, Brown K, Sullivan MD, Borgen P (1996) Perception of breast cancer risk and psychosocial distress in women attending a surveillance program. Psycho-Oncology 5: 259–269

[bib10] Goldberg D, Williams P (1988) A User's Guide to the General Health Questionnaire. Windsor: NFER-Nelson

[bib11] Hopwood P, Shenton A, Lalloo F, Evans D, Howell A (2001) Risk perception and cancer worry: an exploratory study of the impact of genetic risk counselling in women with a family history of breast cancer. J Med Genet 38: 139–142 11288719 10.1136/jmg.38.2.139 PMC1734804

[bib12] Horowitz M, Wilner N, Alvarez W (1979) Impact of Event Scale: a measure of subjective stress. Psychom Med 41: 209–218 10.1097/00006842-197905000-00004 472086

[bib14] Kash K, Holland J, Miller D, Osborne M (1999) Intervention for women at risk for breast cancer. Psycho-Oncology 8(6): 9 (abstract)

[bib13] Kash KM, Holland JC, Halper MS, Miller DG (1992) Psychological distress and surveillance behaviours of women with a family history of breast cancer. J Natl Cancer Inst 84: 24–30 1738170 10.1093/jnci/84.1.24

[bib15] Lerman C, Daly M, Sands C, Balshem A, Lustbader E, Heggan T, Goldstein L, James J, Engstrom P (1993) Mammography adherence and psychological distress among women at risk for breast cancer. J Natl Cancer Inst 85(13): 1074–1080 8515494 10.1093/jnci/85.13.1074

[bib16] Lerman C, Kash K, Stefanek M (1994) Younger women at increased risk for breast cancer: perceived risk, psychological well being and surveillance behavior. J Natl Cancer Inst Monogr 16: 171–176 7999461

[bib17] Lerman C, Schwartz MD, Miller SM, Daly M, Sands C, Rimer BK (1996) A randomized trial of breast cancer risk counseling: interacting effects of counseling, education level, and coping style. Health Psychol 15(2): 75–83 8681923 10.1037//0278-6133.15.2.75

[bib18] Lerman C, Trock B, Rimer BK (1991a) Psychological side effects of breast cancer screening. Health Psychol 10(4): 259–267 1915212 10.1037//0278-6133.10.4.259

[bib19] Lerman C, Trock B, Rimer BK, Boyce A, Jepson C, Engstrom PF (1991b) Psychological and behavioral implications of abnormal mammograms. Ann Intern Med 114: 657–661 2003712 10.7326/0003-4819-114-8-657

[bib20] Lloyd S, Watson M, Waites B, Meyer L, Eeles R, Ebbs S, Tylee A (1996) Familial breast cancer: a controlled study of risk perception, psychological morbidity and health beliefs in women attending for genetic counselling. Br J Cancer 74: 482–487 8695370 10.1038/bjc.1996.387 PMC2074635

[bib21] Marteau T, Lerman C (2001) Genetic risk and behavioural change. BMJ 322: 1056–1059 11325776 10.1136/bmj.322.7293.1056 PMC1120191

[bib22] Pocock SJ (1983) Clinical Trials – a Practical Approach, pp 76–79. Chichester: Wiley & Sons

[bib23] Schwartz MD, Lerman C, Audrain J, Cella D, Rimer B, Stefanek M, Garber J, Lin TH, Vogel V (1998) The impact of a brief problem-solving training intervention for relatives of recently diagnosed breast cancer patients. Ann Behav Med 20(1): 7–12 9755346 10.1007/BF02893803

[bib24] Scottish Cancer Group & Cancer Genetics Sub-Group (2001) Cancer Genetics Services in Scotland: Guidance to Support the Implementation of Genetics Services for Breast, Ovarian and Colorectal Cancer Predisposition. Scottish Executive: Edinburg

[bib25] Taylor SE, Lichtman RR, Wood JV (1984) Attributions, beliefs about control and adjustment to cancer. J Pers Soc Psychol 46(3): 489–502 6707865 10.1037//0022-3514.46.3.489

[bib26] Watson M, Duvivier V, Wade Walsh M, Ashley S, Davidson J, Papaikonomou M, Murday V, Sacks N, Eeles R (1998) Family history of breast cancer: what do women understand and recall about their genetic risk? J Med Genet 35(9): 731–738 9733031 10.1136/jmg.35.9.731 PMC1051425

[bib27] Wellisch D, Hoffman A, Goldman S, Hammerstein J, Klein K, Bell M (1999) Depression and anxiety symptoms in women at high risk for breast cancer: pilot study of a group intervention. Am J Psychiatry 156(10): 1644–1645 10518180 10.1176/ajp.156.10.1644

